# Detection and comparison of microRNAs in the caprine mammary gland tissues of colostrum and common milk stages

**DOI:** 10.1186/s12863-017-0498-2

**Published:** 2017-05-02

**Authors:** Jinxing Hou, Xiaopeng An, Yuxuan Song, Binyun Cao, Heping Yang, Zhou Zhang, Wenzheng Shen, Yunpu Li

**Affiliations:** 1 Animal Engineering Branch, Yangling Vocational & Technical College, No. 10 Xinong Road, Yangling, Shaanxi 712100 People’s Republic of China; 2 College of Animal Science and Technology, Northwest A&F University, No. 22 Xinong Road, Yangling, Shaanxi 712100 People’s Republic of China

**Keywords:** Mammary gland, Dairy goats, Colostrum, Solexa sequencing

## Abstract

**Background:**

MicroRNAs (miRNAs) have a great influence on various physiological functions. A lot of high-throughput sequencing (HTS) research on miRNAs has been executed in the caprine mammary gland at different lactation periods (common milk lactation and dry period), but little is known about differentially expressed miRNAs in the caprine mammary gland of colostrum and peak lactation periods.

**Result:**

This study identified 131 differentially expressed miRNAs (*P* < 0.05 and log_2_ colostrum normalized expression (NE)/peak lactation NE > 1 or log_2_ colostrum NE/peak lactation NE < −1), including 57 known miRNAs and 74 potential novel miRNAs in the colostrum and peak lactation libraries. In addition, compared with differentially expressed miRNAs in the peak lactation period, 45 miRNAs in the colostrum lactation period were remarkably upregulated, whereas 86 miRNAs were markedly downregulated (*P* < 0.05 and log_2_ colostrum NE/peak lactation NE > 1 or log_2_ colostrum NE/peak lactation NE < −1). The expressions of 10 randomly selected miRNAs was analyzed through stem-loop real-time quantitative PCR (RT-qPCR). Their expression patterns were the same with Solexa sequencing results. Pathway analysis suggested that oestrogen, endocrine, adipocytokine, oxytocin and MAPK signalling pathways act on the development of mammary gland and milk secretion importantly. In addition, the miRNA-target-network showed that the bta-miR-574 could influence the development of mammary gland and lactation by leptin receptor (LEPR), which was in the adipocytokine signalling pathway. Chr5_3880_mature regulated mammary gland development and lactation through Serine/threonine-protein phosphatase (PPP1CA), which was in the oxytocin signalling pathway.

**Conclusions:**

Our finding suggested that the profiles of miRNAs were related to the physiological functions of mammary gland in the colostrum and peak lactation periods. The biological features of these miRNAs may help to clarify the molecular mechanisms of lactation and the development of caprine mammary gland.

**Electronic supplementary material:**

The online version of this article (doi:10.1186/s12863-017-0498-2) contains supplementary material, which is available to authorized users.

## Background

The mammary glands of adult female goats have the ability to go through continual developmental series which are regulated by the endocrine system. The changes that happen during lactation, pregnancy and involution have been defined as Lactogenesis I (differentiation, growth and colostrogenesis), Lactogenesis II (feracious milk production) and involution (degradation to a nonlactating state). The pregnancy that induce the changes of endocrine can stimulate the proliferation of mammary epithelial cells which will prepare for feracious milk production (Lactogenesis II) during Lactogenesis I [1, 2]. The initial secretion of mammary gland near or at the time of parturition is defined as colostrum. It occurs in the time of a particular physiological and functional stage of mammary gland development which is significantly distinct from the gland’s primary role of milk production [3]. Colostrum that is secreted initially by mammary gland after parturition differs markedly from mature milk. Colostrum is characterized by high fat as well as protein and mineral contents compared with mature milk. It also presents some interesting components from a biological viewpoint, such as protective substances (immunoglobulins, lactoferrin and lysozymes) and growth factors (vitamins and amino acids) among others [4]. Immunoglobulins are key components that are necessary for the survival of the kid. These immunoglobulins are delivered to the neonate via sucking and afford passively acquired immunity [5, 6].

The mammary gland is the organ in which biosynthesis and secretion of milk take place. These processes involve complex molecular mechanisms for which the regulation remains unknown. Many studies are being conducted aiming to identify the factors that can modulate the composition of milk to meet the demand of the industry and consumers as well as to identify the underlying mechanisms of action and regulation, as precisely as possible [7, 8]. Recent studies have indicated that some miRNAs may have been critical factors for the development of mammary gland via regulating gene expression. MiRNAs are endogenous single-stranded noncoding RNAs (approximately 18–25 nucleotides) that can regulate gene expression through facilitating the degradation or translational repression of target mRNAs via binding to their 3′-untranslated regions [9–11]. In animals, miRNAs are among the most affluent classes of regulators because miRNA genes may regulate up to 60% of protein-coding genes, but only account for 2 to 5% of the total number of all mammalian genes [12–14]. In mouse, over-expression miR-205 increased cellular proliferation and resulted in an expansion of the progenitor-cell population [15]. Over-expression miR-101a suppressed the expression level of *β-casein* mRNA, a marker of cell differentiation and a milk protein, but its suppression was not regulated by direct post-transcriptional or transcriptional regulation of *β-casein* mRNA [16]. Kayo et al. (2014) suggested that the miRNA-132/212 family are essential for the regulation of epithelial duct outgrowth during the development of mice mammary gland [17].

Goats are not only vital livestock animals but also important model organisms for the study of mammary gland bioreactor. Previous studies have identified the profiles of miRNA in the mammary glands of the Guanzhong and Laoshan dairy goats during lactation period via HTS [18–20]. Colostrogenesis is distinct from lactation. However, studies on the miRNA profiles during colostrogenesis were never reported in dairy goats. Furthermore, because of the development of a HTS technology, new goat miRNA data was presented in the miRBase database. So it is necessary to identify the miRNAs that are involved in colostrogenesis and compare the miRNA expression profiles with lactation to screen the novel and differentially expressed miRNAs and illuminate the regulatory mechanisms that are related to the lactating mammary gland. This work would remarkably improve our understanding of the lactating mechanisms of mammary gland.

## Results

### Identification of miRNAs by HTS

**Fig. 1:**
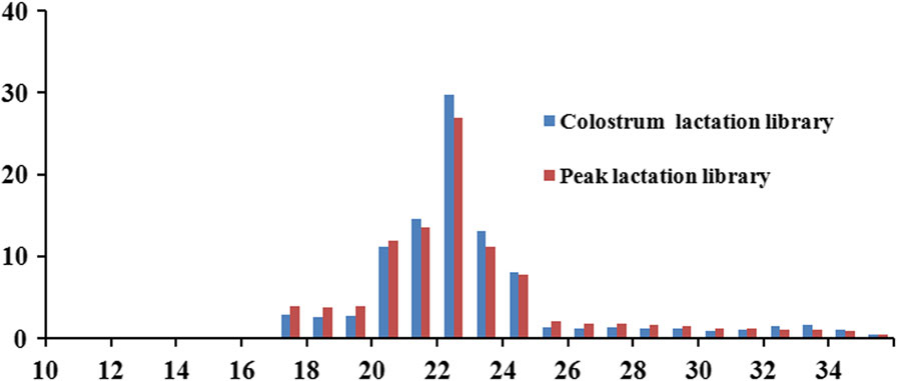
Length distribution and abundance of small RNAs in the colostrum and peak lactation libraries

**Fig. 2:**
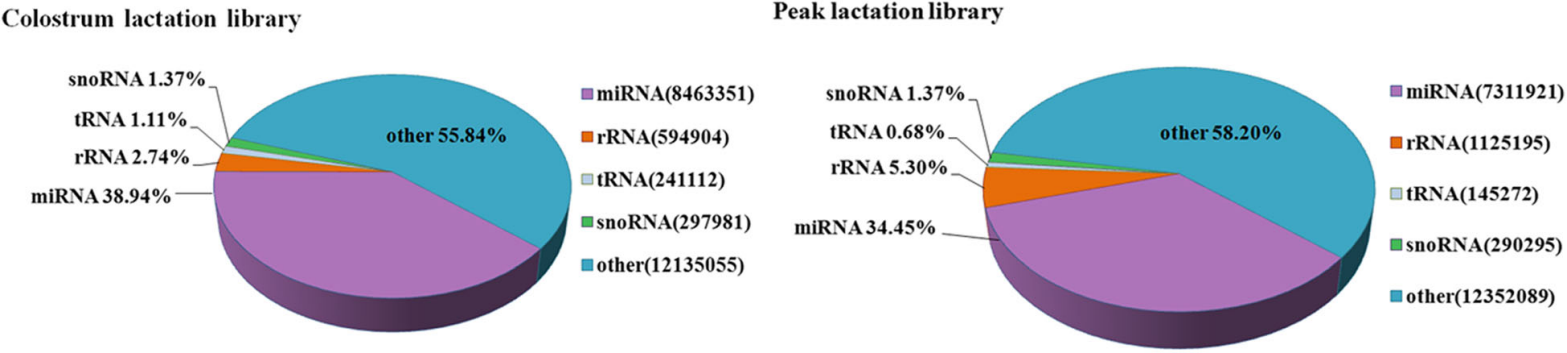
Distribution of small RNAs among different categories in the colostrum and peak lactation libraries. The clean reads were annotated and classified as miRNA, rRNA, tRNA and snoRNA in GenBank and Rfam databases. Partial reads were not annotated

Two small RNA sequencing libraries were prepared for HTS to confirm differentially expressed miRNAs in the caprine mammary gland of colostrum and peak lactation. A total of 12,082,377 and 12,302,426 clean reads were eventually acquired from the colostrum and peak lactation mammary gland tissue libraries, respectively, and then all sequence reads identified were incorporate to predigest the sequencing data. The size distribution of small RNAs was similar between the both libraries. The lengths of the largest number of small RNAs were 20–24 nt. The most affluent size class was 22 nt in the small RNA sequence distribution (Fig. 1), which covered around 29.73 and 26.95% in the colostrum and peak lactation mammary gland tissues, respectively, and followed by 21 nt (14.65%, 13.53%), 23 nt (13.07%, 11.15%) and 20 nt (11.12%, 11.97%), which are the same with the known 18–25 nt range for miRNAs and typical of small RNA Dicer-processed products. According to small RNA annotations, they were divided into several different categories to evaluate the efficiency of HTS for small RNA detection. The tRNA, rRNA, snoRNA and snRNA sequences were removed, which were confirmed though a Basic Local Alignment Search Tool (BLAST) against the known noncoding RNAs that were deposited in the NCBI GenBank and Rfam databases. Small RNA tags were aligned to introns and exons of mRNA to discover the degraded fragments of mRNA and repeat-associated RNA to discover matched tags in the sample. Our results showed that reads of miRNAs were 8,463,351 and 7,311,921, which accounted for 38.94 and 34.45% in the colostrum and peak lactation libraries (Fig. 2), respectively.

### Conserved and novel miRNAs

To confirm conserved and novel miRNAs in the caprine mammary gland, the data was compared with conserved mammalian miRNAs (mature miRNAs and miRNA precursors) in miRBase 21.0 (http://www.mirbase.org/). Sequencing reads that did not match any of conserved miRNAs were further analyzed to find novel miRNAs. One or two mismatches were allowed between sequences, 568 conserved miRNAs were confirmed in the colostrum and peak lactation libraries (Additional file 1: Table S1). A total of 381 potential novel miRNAs have the typical miRNA stem-loop secondary structure (Additional file 2: Table S2), which can form the Dicer enzyme cleavage site. In the colostrum and peak lactation libraries, the chi-miR-143-3p was overwhelmingly expressed with more than 150,000 NE. The chi-miR-30a-5p, chi-miR-148a-3p, chi-miR-26a-5p and chi-miR-10b-5p were overwhelmingly expressed with more than 50,000 NE in both lactation libraries. The expressions of these miRNAs predominates, suggesting that they may have an effect on caprine milk performance.

### Differentially expressed miRNAs

According to the changes of relative miRNA abundance between the both libraries, we screened 131 differentially expressed miRNAs (*P* < 0.05 and log_2_ colostrum NE/peak lactation NE > 1 or log_2_ colostrum NE/peak lactation NE < −1), including 57 known miRNAs and 74 potential novel miRNAs in the colostrum and peak lactation libraries (Additional file 3: Table S3). Compared with the peak lactation, 45 miRNAs in the colostrum lactation were markedly upregulated, whereas 86 miRNAs were markedly downregulated (*P* < 0.05 and log_2_ colostrum NE/peak lactation NE > 1 or log_2_ colostrum NE/peak lactation NE < −1). Among the upregulated miRNAs in the colostrum lactation, chi-miR-223-3p had the most fold change of at least 88 fold, and followed by chi-miR-223-5p with a fold change of at least 72 fold.

### Identification of miRNA expression using stem-loop RT-qPCR

**Fig. 3:**
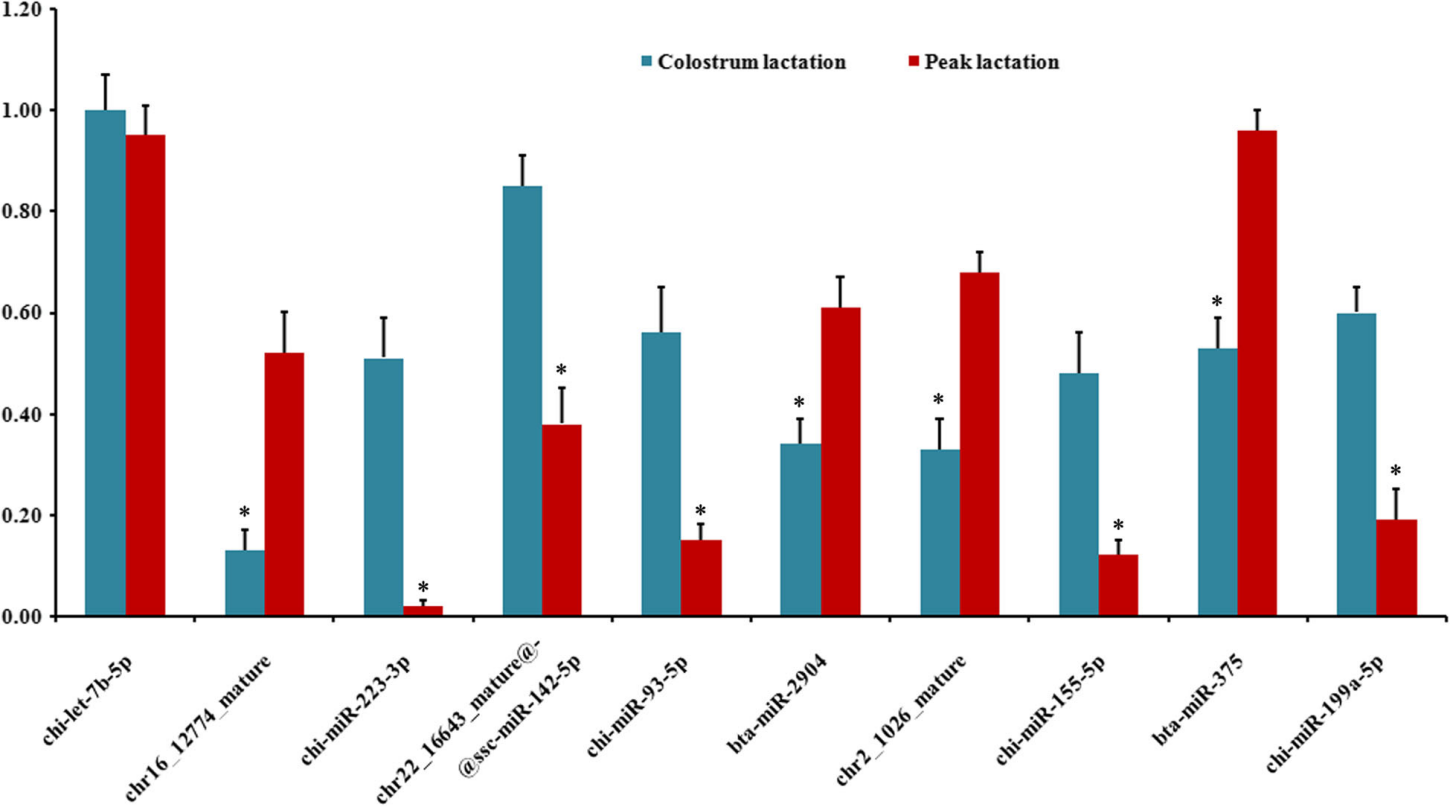
Real-time quantitative PCR validation of identified miRNAs using Solexa sequencing technology. The relative quantification of expression was calculated using the 2^-∆∆Ct^ method after the threshold cycle (Ct) and was normalized with the Ct of 18S rRNA. *represents *P* < 0.05

Stem-loop RT-qPCR was used in the comparison among the expressions of differentially expressed miRNAs to identify the reliability of HTS data. The expressions of ten differentially expressed miRNAs selected randomly were verified in the caprine mammary gland of colostrum and peak lactation. The results are shown in Fig. 3. The expressions of bta-miR-375, chr16_12774_mature, bta-miR-2904 and chr2_1026_mature in the colostrum lactation were lower than those of peak lactation (*P* < 0.05). In addition, compared with the peak lactation, the expressions of chr22_16643_mature@@ssc-miR-142-5p, chi-miR-223-3p chi-miR-93-5p, chi-miR-155-5 and chi-miR-199a-5p were greater in the colostrum lactation (*P* < 0.05). The chi-let-7b-5p was not differentially expressed in the colostrum and peak lactation (*P* > 0.05). The expression patterns were the same with the HTS results.

### MiRNA target gene prediction, GO and KEGG analysis

To further explain the biological processes and physiological functions of the miRNAs screened during the development and lactation of mammary gland, prediction of miRNA target genes was carried out based on mRNA/miRNA interactions to afford some molecular insight into the processes. We used the Targetscan as a tool for predicting the target genes of differentially expressed miRNAs. One miRNA could adjust the expressions of many genes, such as chr4-3031-star, chr15-12382-mature and bta-miR-574. Few miRNAs, which predicted one target gene such as chi-miR-17-5p and chr5-3880-mature, also exist. In addition, many miRNAs were able to co-regulate the expression of one gene (Additional file 4: Figure S1). To functionally categorize miRNA target genes, GO terms were assigned to each differ-gene. Among all items with a *P* ≤ 0.01, 17 items were involved in biological process, 8 items were related with cellular component and 7 items were involved in molecular function (Additional file 5: Figure S2). Pathway analysis was applied to identify the remarkable pathways of the differential genes based on KEGG database. A total of 267 pathways were identified, among which 35 pathways were significantly enriched (*P* < 0.05). Some pathways, such as oestrogen signalling pathway, endocrine, other factor-regulated calcium reabsorption, adipocytokine signalling pathway, oxytocin signalling pathway and MAPK signalling pathway have important parts in mammary development and milk secretion (Additional file 6: Figure S3). In addition, pathway analysis suggested that bta-miR-574 could adjust the development and lactation of mammary gland through LEPR, which was in the adipocytokine signalling pathway. Chr5_3880_mature could adjust the development and lactation of mammary gland through PPP1CA, which was in the oxytocin signalling pathway.

## Discussion

Physico-chemical characterisations of colostrum from human [21], goat [22] and cow breeds [23] have been reported. Common milk is different from colostrum, not only in terms of the composition but also synthesis. Colostrum contains high concentrations of nutrients, such as vitamins, proteins, enzymes, minerals, antimicrobial peptides and immunoglobulins. The process of forming colostrum is called colostrogenesis [2], which can be induced by circulating hormones (estradiol/progesterone) produced by the conceptus or can be artificially induced by injections of hormones. However, the synthesis mechanisms of colostrum are complex. Colostrum is a complex body fluid. Its composition is regulated by hormones (estradiol and progesterone) during colostrogenesis that may be described as the prepartum transfer of components, and this process stop suddenly immediately before parturition [24]. Colostrum components are secreted by different mechanisms. For example, proteins are directly produced in the mammary gland or transferred from the bloodstream, whereas part of the IgA is synthesized by the plasma cells of mammary gland, which migrated into the gland, and IgG is transported to the mammary gland from bloodstream [25].

Following the development of research on the lactating mechanisms of mammary gland, more researchers focus on the action of miRNAs on the post-transcriptional level in various physiological and developmental processes [26]. MiRNA expression profiles in the common milk lactation and dry period have been reported in dairy goats [27, 28]. The function of miRNAs is eventually defined by their effect on the expressions of target genes. Sass et al. (2015) found that the functional roles of miRNA could be finished via functional annotation of target genes and identification of mRNA/miRNA interactions [29]. Ji et al. (2015) showed that prolactin receptor is a target gene of miR-135a that may play a key role in regulating the development and lactation of caprine mammary gland [30]. MiR-143 is a crucial post-transcription regulator that is related to caprine mammary cell survival, and it may have an effect on the development, lactation or involution of mammary gland [31]. Dong et al. (2013) suggested that miR-423-5p, miR-378 and miR-7 could have important regulatory functions during lactation with respect to milk ingredient transport and milk ingredient synthesis in the mammary gland [27]. Giles et al. (2013) showed that insulin receptor substrate-2 (IRS-2) is a target gene of miR-7-5p and an activator of protein kinase B (Akt), which has important functions in glycometabolism and protein synthesis [32]. Estrogen is important for milk secretion, and Xu et al. (2011) reported that estradiol production is posttranscriptionally downregulated by miR-378 [33]. Lin et al. (2013) found that over-expression miR-27a decreased triglyceride via suppressing peroxisome proliferator-activated receptor gamma (PPARγ) protein levels [34]. In this study, chr4-3031-star, chr15-12382-mature and bta-miR-574 regulated the expressions of many target genes. Chi-miR-17-5p and chr5-3880-mature affected one target gene. In addition, chr2-1153-mature and chr15-12204-mature were able to co-regulate the expression level of one gene. According to our study, the GO enrichment revealed that the target genes of some miRNAs were referred to cellular processes, intracellular component ontology and binding functions. KEGG pathway analysis suggested that the genes were devoted to oestrogen, endocrine, other factor-regulated calcium reabsorption, adipocytokine, oxytocin and MAPK signalling pathways. For example, in the adipocytokine signalling pathway, bta-miR-574 might adjust the development and lactation of mammary gland through LEPR. In the oxytocin signalling pathway, chr5_3880_mature might regulate the development and lactation of mammary gland by PPP1CA. These results showed that some miRNAs might be referred to mammary gland cell differentiation, proliferation and apoptosis, and the lactation and physiology of mammary gland.

## Conclusions

This present study confirmed 57 known miRNAs and 74 potential novel miRNAs that are significantly different in the colostrum and peak lactation libraries. In the colostrum lactation library, 45 miRNAs were significantly upregulated, whereas 86 miRNAs were significantly downregulated compared with the peak lactation library. KEGG analysis showed that the target genes of some miRNAs were referred to the oestrogen, oxytocin, MAPK signalling pathways, etc. According to the miRNA-target-network, bta-miR-574 and Chr5_3880_mature might especially important regulatory functions in mammary gland biology during lactation regarding milk ingredient transport and ingredient synthesis. Our results afforded an in-depth understanding on the miRNA mediated regulation of target genes in the physiological function of lactating mammary gland.

## Methods

### Sample collection and RNA extraction

Dairy goats came from Fuping Goat Breeding Center, in Fuping county of Shaanxi province, China. The breast tissues were collected at the colostrum (2 days postpartum) and peak lactation (90 days postpartum) periods from 18 healthy Guanzhong dairy goats (3-year-olds) by surgery. All collected tissues were quickly frozen in liquid nitrogen and stored at −80 °C. TRIzol reagent was used to extract total RNAs according to its instructions (Invitrogen, Carlsbad, USA), and then total RNAs were homogenized and pooled for HTS. The purity and concentration of total RNAs were detected with an Epoch microplate spectrophotometer (BioTek Instruments, Inc., USA). The OD_260/280_ ratios were >1.8 and <2.1 for all samples.

### HTS of colostrum and peak lactation libraries

The homogenized RNAs of nine dairy goats were mixed, and then divided into three groups to perform HTS for colostrum library. The same method was used to construct the peak lactation library. Briefly, polyacrylamide gel electrophoresis (PAGE) was used to purify the total RNAs for enriching 18–30 nt molecules. Subsequently, the special adapters were joined to the 5′ and 3′ end of the RNAs with T4 RNA ligase, and the treated RNA as templates synthesized cDNA. The libraries for HTS were generated through amplifying the cDNA with PCR. The amplified products were purified with 4% agarose gels, and then the purified products were used for Solexa sequencing on an Illumina Genome Analyzer (Illumina, SanDiego, USA) at LC Sciences (Houston, Texas, USA). The digital-quality data were generated via processing the image files produced by the sequencer. The subsequent procedures that were carried with Solexa generalized the data production, assessed the quality of sequencing, calculated the length of small RNA reads, and filtrated reads that were contaminated by tRNA, rRNA, snRNA, snoRNA and mRNA. In the end, clean reads were aligned to a miRBase database (release 21.0).

### Bioinformatics analysis of sequencing data

The raw data were handled to acquire clean reads via removing low quality reads, reads with 5' primer contaminants, reads without 3'primer, reads with poly(A), reads without the inserted tag, and reads shorter than 18 nt. The clean reads were aligned to *Capra hircus* and other mammal genomes through SOAPv1.11 Software to analyze their distribution and expression. The matched sequences were mapped to the NCBI GenBank (http://blast.ncbi.nlm.nih.gov/) and Rfam database (http://rfam.janelia.org) to confirm and eliminate the snRNA, tRNA, scRNA, rRNA, snoRNA and srpRNA sequences, and then the residual sequences were blasted against miRBase 21.0 database. Sequences in the libraries with related (permitting one or two nucleotide substitutions) or identical sequences to *Capra hircus* or other mammals (*Ovis aries, Bos taurus, Sus scrofa, Homo sapiens, Equus caballus* and *Canis familiaris*) were defined as known miRNAs. Sequences that were not mapped to any of the conserved miRNAs were further explored to find novel miRNAs. This study analyzed their dicer cleavage sites, hairpin structures and minimal free energies through MIREAPv0.2 Software to identify whether these sequences were real goat miRNAs [35]. In addition, we used MiPred and Mfold Softwares to predict the secondary structures of miRNA precursors and eliminate pseudo-pre-miRNAs [36–38].

### Differential expression miRNAs and target gene prediction

The abundances of differential expression miRNAs were normalized to gain the expressions of transcripts per million in the caprine mammary gland of colostrum and peak lactation. Normalized expression (NE) values of zero between the both libraries were modified to 0.001. Samples with NE values of less than three in both libraries were not analyzed further. The *P*-value and fold-change were calculated from the NE with the following formulas:

$$ \mathrm{N}\mathrm{E} = \left(\mathrm{Factual}\ \mathrm{miRNA}\ \mathrm{number}/\mathrm{Total}\ \mathrm{number}\ \mathrm{of}\ \mathrm{clean}\ \mathrm{reads}\right) \times 1000000 $$

$$ \mathrm{Fold}\ \mathrm{change} = {\mathrm{Log}}_2\left(\mathrm{colostrum}\ \mathrm{NE}/\mathrm{peak}\ \mathrm{lactation}\ \mathrm{NE}\right) $$

*P*-value:

$$ P\left( x\Big| y\right)=\left(\frac{N_2}{N_1}\right)\underset{D\left( y\ge {y}_{\min}\Big| x\right)={\displaystyle \sum_{y\ge {y}_{\max}}^{\infty } p\left( y\Big| x\right)}}{\overset{C\left( y\le {y}_{\min}\Big| x\right)={\displaystyle \sum_{y=0}^{y\le {y}_{\min }} p\left( y\Big| x\right)}}{\frac{\left( x+ y\right)}{x! y!{\left(1+\frac{N_2}{N_1}\right)}^{\left( x+ y+1\right)}}}} $$

The *x* and *y* values in formulas stand for the NE level, and the *N*_1_ and *N*_2_ values stand for the total number of clean reads of a given miRNA in the libraries of the mammary gland of colostrum and peak lactation, respectively. According to the criteria of the Fold change > 2 or < 0.5 and *P*-value < 0.05, FDR < 0.05 [39], the differentially expressed miRNAs were selected from the both libraries. The Miranda was used for predicting miRNA targets on the differentially expressed miRNAs.

### Gene ontology (GO) and KEGG analysis of target genes

GO was executed to promote the understanding on the biological function of target genes of the differentially expressed miRNA. We downloaded the GO annotations from the Gene Ontology (http://www.geneontology.org/), NCBI (http://www.ncbi.nlm.nih.gov/) and UniProt (http://www.uniprot.org/). GO enrichment and KEGG pathway analysis were conducted for target genes by hypergeometric test with corrected *P*-value ≤ 0.05. The following formula calculates the *P*-value:

$$ \mathrm{P}=1-{\displaystyle \sum_{i=0}^{m-1}}\frac{\left(\begin{array}{c}\hfill M\hfill \\ {}\hfill i\hfill \end{array}\right)\left(\begin{array}{c}\hfill N- M\hfill \\ {}\hfill n- i\hfill \end{array}\right)}{\left(\begin{array}{c}\hfill N\hfill \\ {}\hfill n\hfill \end{array}\right)} $$

In this study, *N* represents the number of all genes with GO annotations, *n* represents the number of target genes in *N*, *M* represents the number of all genes that are annotated to special GO terms, and *m* represents the number of target genes in *M*.

The KEGG (http://www.genome.jp/kegg/) pathway [40] was analyzed through the ClueGO plug-in (http://apps.cytoscape.org/apps/cluego) [41] and Cytoscape software V2.8.2 (http://www.cytoscape.org/) [42] to identify the significant pathways of the differential genes and clarify their biological functions. The formula was consistent with that of GO annotations, but *N* represents the number of all genes with KEGG analysis, *n* represents the number of target genes in *N*, *M* represents the number of all genes annotated to special pathways, and *m* represents the number of target genes in *M*.

### MiRNA-target-network construction

According to the differential expression values of miRNAs and target genes, their interactions in Sanger miRNA database were analyzed to construct the miRNA- target-network. The adjacency matrix of genes and miRNAs A = [*a*_*i,j*_] is established via the attribute relationships among miRNAs and genes. *a*_*i,j*_ represents the relative weight of gene *i* and miRNA *j*. In the miRNA-target-network, the circle means the gene, the square means miRNAs and their relationship is showed via one edge. The network centre is represented via a degree. Degree represents the donation of one miRNA to the genes around or the donation of one gene to the miRNAs around. The critical miRNAs and genes in the target network have the highest degrees based on the NCBI protein-protein-interaction database.

### Identification of miRNAs with stem-loop RT-qPCR

Ten randomly selected miRNAs were validated with stem-loop RT-qPCR in the two libraries. The system and program of reverse transcription reaction were as follows: (1) RNase-Free dH_2_O was added to the mixture of 0.5 μl of RT primer (2 uM) and 250 ng of RNAs until a final volume of 3 μl. Their mixture was incubated 65 °C for 10 min, and subsequently snapped on ice for 3 min. (2) Denatured RNAs and RT primer (3 μl) were mixed with 1 μl of 5 × RT buffer, 0.25 μl of dNTP (10 mM), 0.5 μl of M-MLV (200 U/μl) and 0.25 μl of RNase inhibitor (40 U/μl), and then the mixture was incubated at 42 °C for 60 min followed by 70 °C for 15 min. The cDNA products were used for RT-qPCR analysis according to Platinum SYBR Green qPCR SuperMix-UDG (Invitrogen, Carlsbad, USA). RT-qPCR program was executed with the ABI PRISM7900HT Real-Time PCR Analyser (ABI, Carlsbad, USA). The PCR mixture was produced through mixing 10 μl of 2 × SYBR Green Mix, 0.8 μl of primer mix (10 uM), 0.5 μl of cDNA for each miRNA, and 8.7 μl of RNase-Free dH_2_O. Thermal cycling conditions were 50 °C for 2 min and at 95 °C for 2 min, followed by 40 cycles at 95 °C for 15 s and at 60 °C for 30 s. 18S rRNA was selected to normalize the expressions of miRNAs. All detection reactions were performed in triplicate. The relative expression levels of miRNAs were calculated with the 2^-∆∆Ct^ method and were standardized with the threshold cycle (Ct) of 18S rRNA. Additional file 7: Table S4 shows the RT-qPCR and reverse transcription primers.

## Additional files

Additional file 1: Table S1. Summary of known miRNAs in the colostrum and peak lactation libraries. (XLSX 50 kb)

Additional file 2: Table S2. Predicted novel miRNAs. (XLSX 35 kb)

Additional file 3: Table S3. Differentially expressed miRNAs in the colostrum and peak lactation libraries. (XLSX 26 kb)

Additional file 4: Figure S1. MiRNA–mRNA network. Red and green box nodes represent miRNAs, and blue cycle nodes represent mRNAs. Edges show the inhibitory effect of miRNAs on mRNAs. (TIF 16083 kb)

Additional file 5: Figure S2. Gene ontology analysis results based on biological process, cellular component, and molecular function. BP: Biological process, CC: Cellular component, and MF: Molecular function. (TIF 15871 kb)

Additional file 6: Figure S3. KEGG pathway annotations for the predicted target genes. (TIF 23133 kb)

Additional file 7: Table S4. Information of primers for reverse transcription and stem-loop qRT-PCR. (DOCX 15 kb)

## Abbreviations

LEPR: Leptin receptor
MiRNAs: MicroRNAs
NE: Normalized expression
PPP1CA: Serine/threonine-protein phosphatase
qRT-PCR: Real-time quantitative PCR
