# Supplementary figures and images for: Detection and comparison of microRNAs in the caprine mammary gland tissues of colostrum and common milk stages
Source: BMC Genet. 2017 May 2;18:38. doi: 10.1186/s12863-017-0498-2 (PMC5414302; doi:10.1186/s12863-017-0498-2)

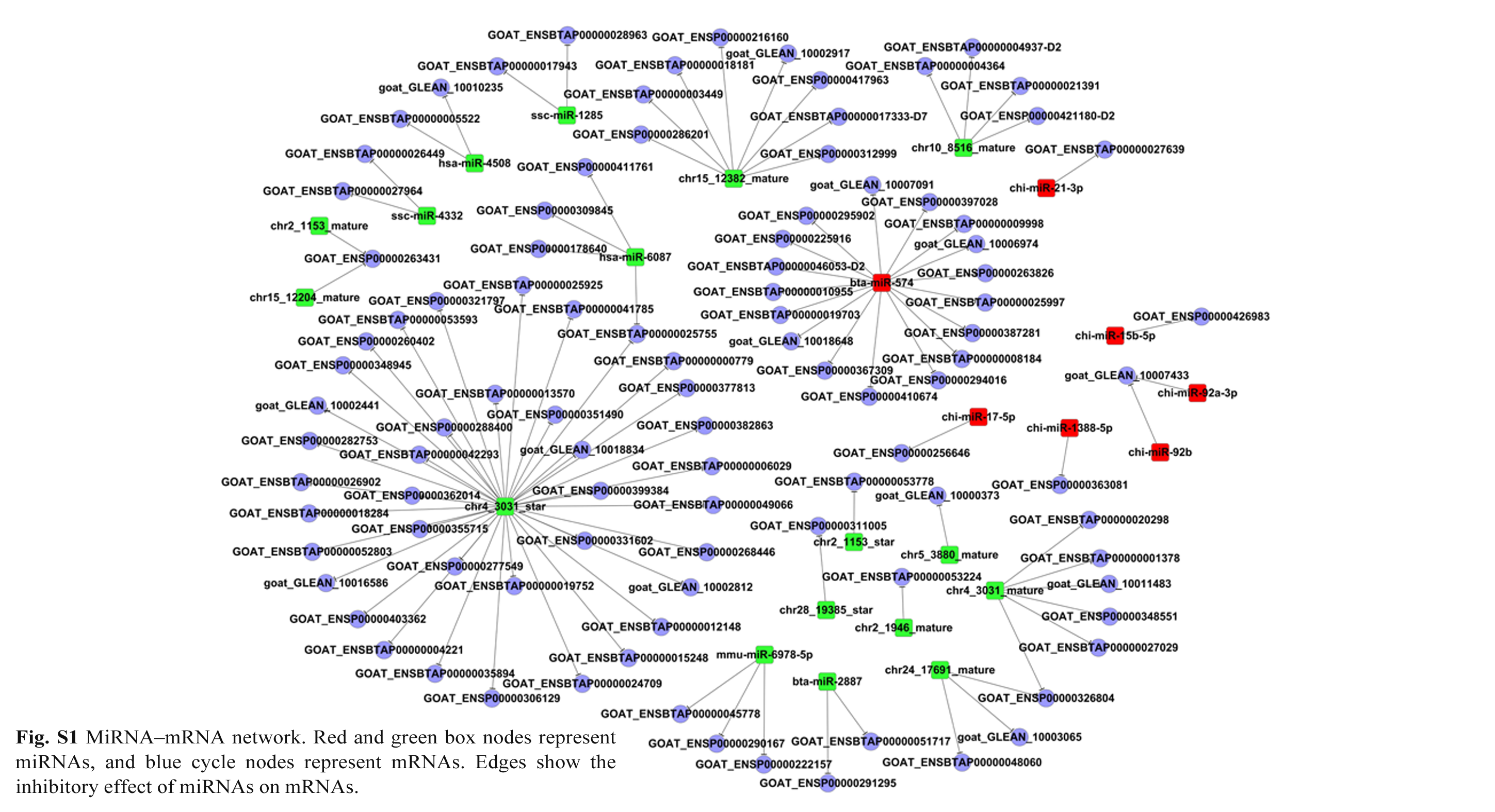

Supplement: Supplementary file 4 — MiRNA–mRNA network. Red and green box nodes represent miRNAs, and blue cycle nodes represent mRNAs. Edges show the inhibitory effect of miRNAs on mRNAs. (TIF 16083 kb) [file 12863_2017_498_MOESM4_ESM.tif]

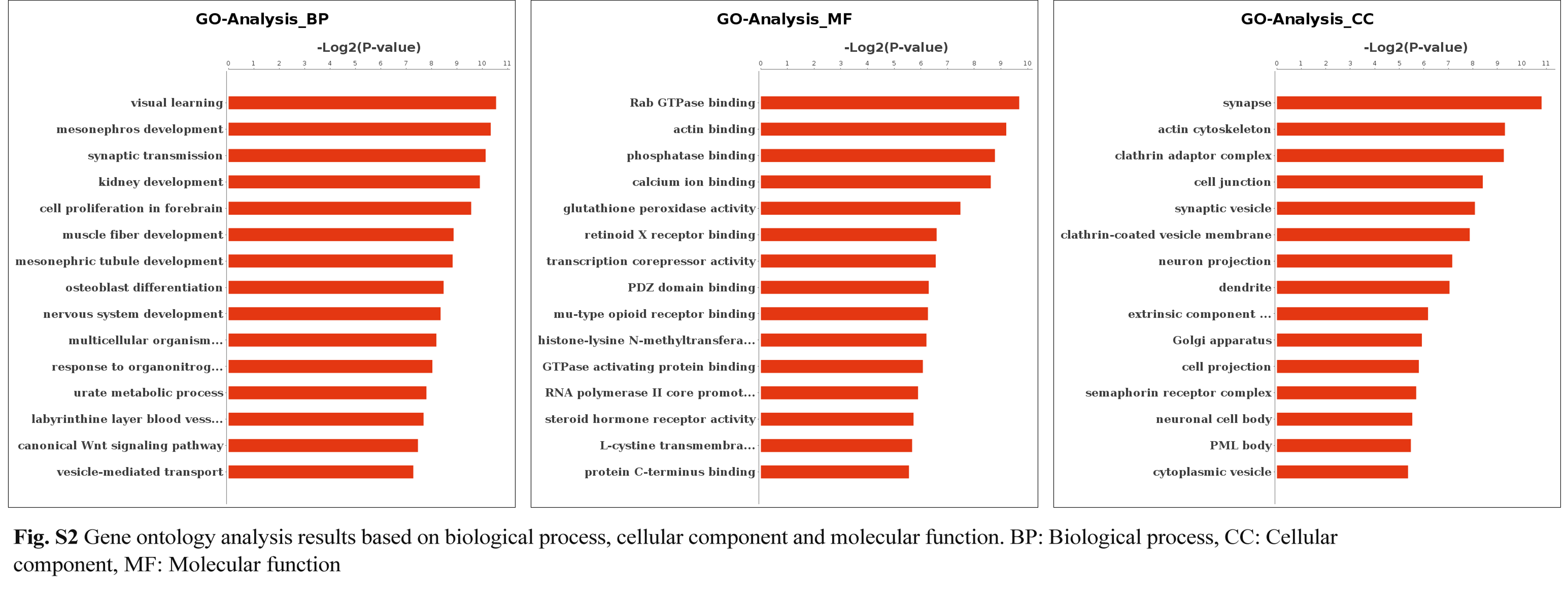

Supplement: Supplementary file 5 — Gene ontology analysis results based on biological process, cellular component, and molecular function. BP: Biological process, CC: Cellular component, and MF: Molecular function. (TIF 15871 kb) [file 12863_2017_498_MOESM5_ESM.tif]

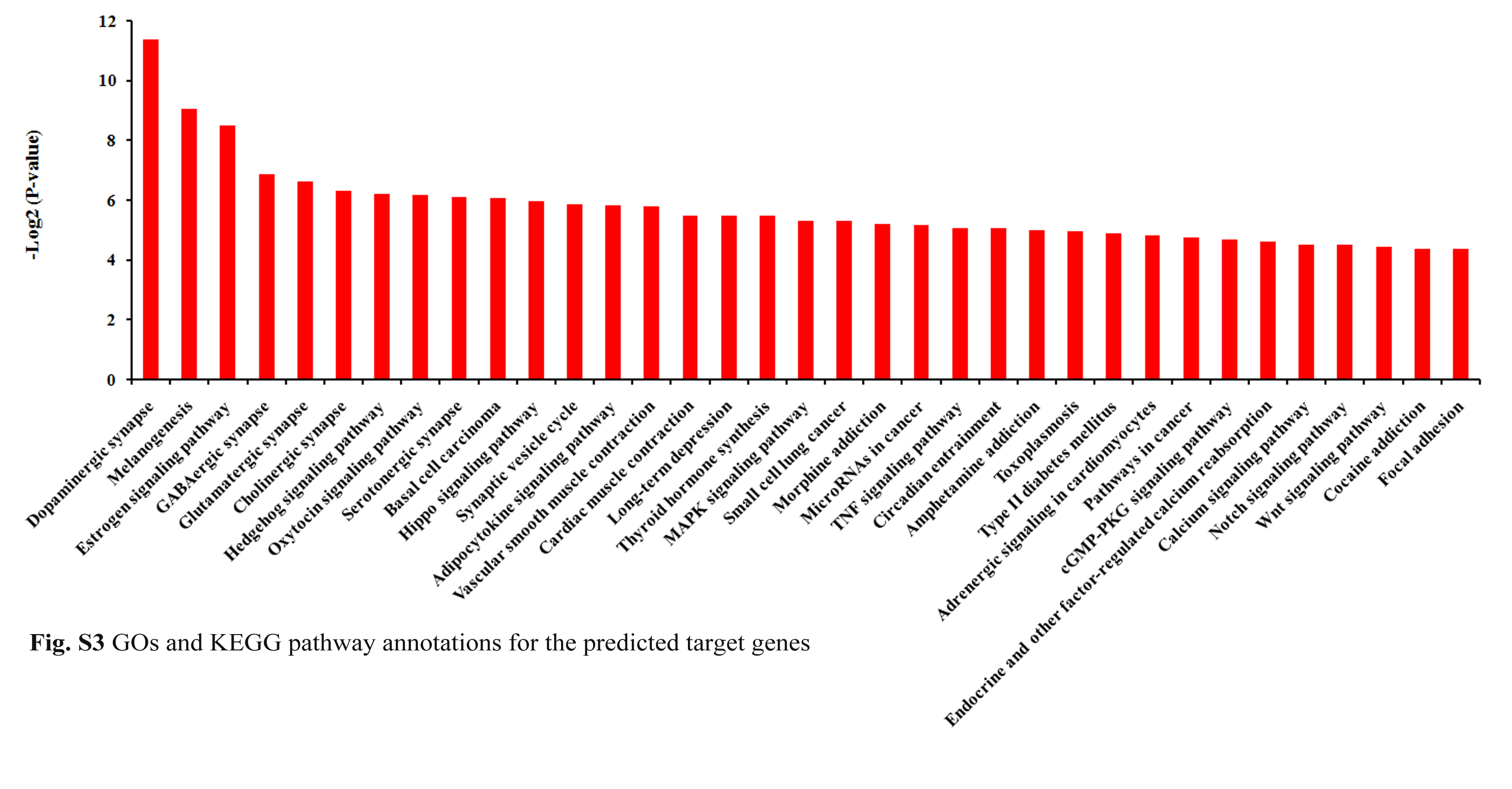

Supplement: Supplementary file 6 — KEGG pathway annotations for the predicted target genes. (TIF 23133 kb) [file 12863_2017_498_MOESM6_ESM.tif]
