# Supplementary material for: Detection and comparison of microRNAs in the caprine mammary gland tissues of colostrum and common milk stages
Source: BMC Genet. 2017 May 2;18:38. doi: 10.1186/s12863-017-0498-2 (PMC5414302; doi:10.1186/s12863-017-0498-2)
Supplement: Supplementary file 7 — Information of primers for reverse transcription and stem-loop qRT-PCR. (DOCX 15 kb) [file 12863_2017_498_MOESM7_ESM.docx]

**Table S1** Information of primers for reverse transcription and stem-loop qRT-PCR

| **miRNA** | **miRNA sequence (5'-3')** | **Primer** | **Primer sequence (5'-3')** | **Length of primer** |
| --- | --- | --- | --- | --- |
| chi-let-7b-5p | tgaggtagtaggttgtgtggtt | Forward prime | TGGCGGTGAGGTAGTAGGTTGT | 22 |
|  |  | RT primer | GTCGTATCCAGTGCAGGGTCCGAGGTATTCGCACTGGATACGACAACCAC |  |
| chi-miR-223-3p | tgtcagtttgtcaaatacccca | Forward primer | GCGGCGGTGTCAGTTTGTCAAAT | 23 |
|  |  | RT primer | GTCGTATCCAGTGCAGGGTCCGAGGTATTCGCACTGGATACGACTGGGGT |  |
| bta-miR-375 | ttttgttcgttcggctcgcgtga | Forward primer | CGGCGGTTTTGTTCGTTCGGC | 21 |
|  |  | RT primer | GTCGTATCCAGTGCAGGGTCCGAGGTATTCGCACTGGATACGACTCACGC |  |
| chr22_16643_mature | agtaatgctttctacttta | Forward primer | CGGCGGCGGAGTAATGCTTTC | 21 |
|  |  | RT primer | GTCGTATCCAGTGCAGGGTCCGAGGTATTCGCACTGGATACGACTAAAGT |  |
| chi-miR-93-5p | caaagtgctgttcgtgcaggtag | Forward primer | GGCGGCAAAGTGCTGTTCGTG | 21 |
|  |  | RT primer | GTCGTATCCAGTGCAGGGTCCGAGGTATTCGCACTGGATACGACCTACCT |  |
| chr2_1026_mature | gaggttatctagagtca | Forward primer | CGGCGGCGGGAGGTTATCTA | 20 |
|  |  | RT primer | GTCGTATCCAGTGCAGGGTCCGAGGTATTCGCACTGGATACGACTGACTC |  |
| bta-miR-2904 | gggagcctcggttggcctc | Forward primer | GGCGGGGGAGCCTCGGTT | 18 |
|  |  | RT primer | GTCGTATCCAGTGCAGGGTCCGAGGTATTCGCACTGGATACGACGAGGCC |  |
| chi-miR-199a-5p | cccagtgttcagactacctgttc | Forward primer | GGCGGCCCAGTGTTCAGACTA | 21 |
|  |  | RT primer | GTCGTATCCAGTGCAGGGTCCGAGGTATTCGCACTGGATACGACGAACAG |  |
| chi-miR-155-5p | ttaatgctaatcgtgataggggt | Forward primer | GCGGCGGTTAATGCTAATCGTGA | 23 |
|  |  | RT primer | GTCGTATCCAGTGCAGGGTCCGAGGTATTCGCACTGGATACGACACCCCT |  |
| chr16_12774_mature | atctttagggtcctaac | Forward primer | CGGCGGCGGATCTTTAGGG | 19 |
|  |  | RT primer | GTCGTATCCAGTGCAGGGTCCGAGGTATTCGCACTGGATACGACGTTAGG |  |
| Reverse primer of Stem-loop qRT-PCR for miRNAs |  | Reverse primer | ATCCAGTGCAGGGTCCGAGG | 20 |
| 18S rRNA |  | Forward primer | GTGGTGTTGAGGAAAGCAGACA | 22 |
|  |  | Reverse primer | TGATCACACGTTCCACCTCATC | 22 |
|  |  | RT primer | TGATCACACGTTCCACCTCATC | 22 |
